# Long-term upadacitinib treatment in a patient with atopic dermatitis, bullous pemphigoid, and alopecia areata: a case report and review of the literature

**DOI:** 10.3389/fmed.2026.1959635

**Published:** 2026-09-30

**Authors:** Sara Triolo, Manfredo Bruni, Lina Maria Magnanimi, Andrea De Berardinis, Simone Mozzato, Mirco Mastrangelo, Cristina Pellegrini, Maria Esposito

**Affiliations:** Department of Biotechnological and Applied Clinical Sciences, University of L'Aquila, L'Aquila, Italy

**Keywords:** alopecia areata, atopic dermatitis, bullous pemphigoid, dupilumab, JAK inhibitors, upadacitinib

## Abstract

Atopic dermatitis (AD), bullous pemphigoid (BP), and alopecia areata (AA) are immune-mediated dermatologic diseases characterized by distinct clinical features but partially overlapping immunopathogenic mechanisms. Their coexistence in a single patient is uncommon and may pose significant diagnostic and therapeutic challenges. We report the case of a 33-year-old woman with long-standing moderate-to-severe AD who subsequently developed BP, confirmed by histopathology and direct immunofluorescence. Initial treatment with systemic corticosteroids and conventional immunosuppressants, including azathioprine followed by cyclosporine, resulted in only transient improvement and was limited by adverse effects. Dupilumab was therefore initiated, leading to marked improvement of BP while maintaining remission of AD. However, after approximately six months of therapy, the patient developed new-onset patchy AA. Dupilumab was discontinued, and treatment was switched to upadacitinib, a selective Janus kinase 1 (JAK1) inhibitor. Early clinical improvement was observed after six weeks, with progressive hair regrowth and stable BP activity. Complete remission of BP was achieved after twelve weeks and complete remission of AA after twenty-four weeks, while remission of AD was maintained. Disease control was sustained throughout a twenty-four -month follow-up, without significant adverse events. This case underscores the diagnostic and therapeutic complexity associated with the coexistence of multiple immune-mediated dermatologic diseases. The sequential emergence of AD, BP, and AA highlights the challenges posed by overlapping inflammatory pathways and the need for alternative therapeutic strategies when pathway-specific treatments prove insufficient or poorly tolerated. These observations support the concept that broader immunomodulatory approaches targeting shared signaling pathways may enable simultaneous control of multiple inflammatory conditions. In this context, inhibition of the JAK–STAT pathway may represent a promising integrated therapeutic strategy in selected patients with complex immune-mediated dermatologic comorbidities.

## 1 Introduction

Atopic dermatitis (AD), bullous pemphigoid (BP), and alopecia areata (AA) are immune-mediated dermatologic disorders with distinct clinical features but partially overlapping immunopathogenic pathways. AD is a chronic eczematous dermatitis characterized by a Th2-skewed immune profile with high levels of interleukin (IL)-4 and IL-13, alongside a disrupted skin barrier (1). BP is an autoimmune subepidermal blistering disorder characterized by IgG autoantibodies against the hemidesmosomal antigens BP180 and/or BP230 and also exhibits a strong type 2 inflammatory signature, with elevated IL-4/IL-13 and IgE levels frequently observed in patients (2). AA, by comparison, is a non-scarring autoimmune hair loss disorder predominantly driven by Th1 pathways, particularly interferon-*γ* and cytotoxic T cells, although recent evidence suggests heterogeneous immune endotypes, including Th1- and Th2-skewed forms, especially in patients with concomitant AD (3).

Epidemiologic studies have demonstrated a bidirectional association between AD and AA, implying shared pathways or genetic susceptibilities (4). Likewise, although the coexistence of AD and BP is infrequently reported, emerging data indicate that a history of AD may increase the risk of developing BP, hinting at an immunologic interplay between these conditions (5).

From a therapeutic standpoint, the management of these conditions has evolved substantially with the introduction of targeted immunomodulatory agents. AD treatment has been transformed by biologic therapies targeting Th2 cytokines, particularly monoclonal antibodies directed against the IL-4 receptor *α* and IL-13. In parallel, given the increasingly appreciated role of type-2 inflammation in BP, dupilumab has recently received FDA approval for BP based on phase 2/3 clinical trial data, while regulatory approval in Europe is still pending (6). In contrast, the treatment paradigm for AA has instead shifted toward Janus kinase (JAK) inhibition, reflecting the key role of interferon and *γ*c-family cytokine signaling in hair follicle autoimmunity (7).

The JAK–STAT pathway represents a shared downstream signaling hub for multiple cytokines implicated across AD, BP, and AA. JAK inhibitors - including upadacitinib and abrocitinib (both JAK1-selective), as well as baricitinib (a JAK1/2 inhibitor)—have received regulatory approval from the EMA and FDA for moderate-to-severe AD (8). For AA, systemic JAK inhibitors such as baricitinib and ritlecitinib (a JAK3/TEC family inhibitor) have been approved for severe disease (9). Although upadacitinib is not currently approved for AA, phase III trials are ongoing and have shown encouraging outcomes (NCT06012240) (10). In BP, JAK inhibition is not yet part of standard clinical practice, although emerging clinical evidence suggests potential efficacy (2, 11).

Against this backdrop, we present a novel case of a young adult patient with a history of AD who subsequently developed BP and, following successful treatment with dupilumab, experienced the onset of AA. This case is clinically and mechanistically noteworthy for its therapeutic and immunological evolution: dupilumab induced sustained remission of BP while effectively controlling AD, but was followed by the paradoxical development of AA. Switching to upadacitinib resulted in remission of AA while maintaining control of both AD and BP. To our knowledge, this is the first reported case of sequential AD, BP, and AA managed with targeted immunomodulatory therapies, highlighting the potential for shared immune pathways to guide integrated treatment of complex dermatologic comorbidities.

## 2 Case presentation

A 33-year-old woman presented to our dermatology unit for rapidly progressive, intensely pruritic bullous eruption affecting the trunk and extremities, requiring hospital admission. Her past medical history included a long-standing history of moderate-to-severe AD, primarily involving the face, neck, and flexural areas, seasonal allergic rhinoconjunctivitis, allergic asthma and celiac disease. Family history was unremarkable for autoimmune or dermatologic diseases. Her AD had previously been managed with topical emollients and corticosteroids, along with occasional short courses of systemic corticosteroids during flares, without achieving adequate disease control.

Physical examination revealed multiple tense bullae arising on an erythematous base, some intact and others eroded. The lesions were diffusely distributed across the trunk and extremities, while mucosal surfaces were spared.

A diagnostic work-up was performed, including lesional and perilesional skin biopsies. Histopathologic examination demonstrated a subepidermal blister associated with a dermal inflammatory infiltrate rich in eosinophils. Direct immunofluorescence revealed linear IgG and C3 deposition along the dermoepidermal junction, with no evidence of IgA or IgM deposition.

Serologic evaluation performed by enzyme-linked immunosorbent assay (ELISA) confirmed the presence of circulating anti-BP180 autoantibodies (118.20 U/mL; reference value <9.0), whereas anti-BP230 autoantibodies were negative (2.26 U/mL; reference value <9.0). Autoantibodies against desmoglein 1 and desmoglein 3 were also negative (0.40 U/mL; reference value <14). Baseline total IgE levels were elevated (719 IU/mL; reference value <100). Additional laboratory investigations were performed to exclude other autoimmune blistering disorders. Anti-endomysial antibodies were absent (indirect immunofluorescence assay). Anti–tissue transglutaminase IgA antibodies were within the normal range (3.1 U/mL; reference range 0–10 U/mL). Anti–deamidated gliadin IgA and IgG antibodies were negative (0 U/mL; reference range 0–10 U/mL). Total IgA levels were within the normal range (332 mg/dL; reference range 70–400 mg/dL). These findings excluded dermatitis herpetiformis. Antinuclear antibodies and extractable nuclear antigen antibodies were negative.

Based on the clinical, histopathologic, immunopathologic, and serologic findings, a diagnosis of BP was established. At the time of diagnosis, disease activity was severe with a Bullous Pemphigoid Disease Area Index (BPDAI) activity score of 180 (erosions/blisters: 115; urticaria/erythema/other: 65; mucosal activity: 0) and a numeric rating scale (NRS) for pruritus of 9.

During hospitalization, the patient received systemic corticosteroid therapy with prednisone at a dose of 0.5–1 mg/kg/day, together with intravenous fluids, and daily local wound care with topical corticosteroid and antibiotics. Partial clinical improvement was observed.

Following discharge, the patient continued on tapering oral prednisone and was started on azathioprine as a steroid-sparing agent at a dose of 100 mg/day. Azathioprine resulted in an initial partial clinical response, with a reduction in new blister formation; however, disease control was not sustained, with recurrence of cutaneous lesions during treatment. After three months, azathioprine was discontinued because of elevated liver transaminases. Treatment with cyclosporine 3 mg/kg/day was then initiated but was discontinued after four weeks due to poor clinical response, particularly persistent severe pruritus.

Given the suboptimal response and treatment-limiting adverse effects of conventional systemic immunosuppressants, treatment with dupilumab was initiated (600 mg subcutaneous loading dose followed by 300 mg every two weeks), with the aim of achieving concomitant control of both AD and BP. At baseline for dupilumab therapy, total IgE levels were 807 IU/mL, BPDAI activity score was 20 (erosions/blisters: 6; urticaria/erythema/other: 14; mucosal activity: 0) and pruritus NRS was 8.

After twelve weeks of dupilumab therapy, the patient showed marked clinical improvement with a substantial reduction in bullous disease activity with a BPDAI activity score of 15, comprising 2 points for erosions/blisters and 13 for urticaria/erythema, with no mucosal involvement, and a pruritus NRS of 4. Residual post-inflammatory hyperpigmentation persisted.

Approximately twenty-four weeks into dupilumab therapy, the patient developed patchy hair loss on the scalp. On examination, non-scarring alopecic patches were noted on the parietal and occipital scalp, with exclamation mark hairs at the periphery and no signs of inflammation or scarring. The findings were consistent with patchy AA, with a Severity of Alopecia Tool (SALT) score of 58. At that time, BP activity was minimal, with a BPDAI activity score of 2, entirely attributable to urticaria/erythema, with no erosions/blisters or mucosal involvement. The patient had no prior history of AA.

In response to the new diagnosis, dupilumab was discontinued and treatment with upadacitinib, a selective JAK1 inhibitor, was initiated at a dose of 15 mg orally once daily. Six weeks after treatment initiation, early clinical improvement was observed, with partial hair regrowth and a reduction in disease activity (SALT score 46), while BP activity remained stable (BPDAI activity score 2). After twelve weeks of treatment, the patient showed marked hair regrowth in the alopecic patches, with the SALT score improving from 58 to 5. At the same time, the patient remained free of bullous lesions, with complete remission of BP (BPDAI activity score 0), and without residual eczematous dermatitis. Pruritus had completely resolved, with a pruritus NRS of 0. At twenty-four weeks of treatment, the patient demonstrated sustained complete remission of BP (BPDAI activity score 0), excellent control of AD (Eczema Area and Severity Index (EASI) score 0; pruritus NRS 0), and complete hair regrowth (SALT score 0).

At the most recent follow-up, twenty-four months after initiation of upadacitinib, the patient demonstrated complete remission of AD, BP and AA. She continues treatment with upadacitinib under regular clinical monitoring, with sustained disease control and no evidence of relapse or significant adverse events (Figures 1–3).

**Figure 1:**
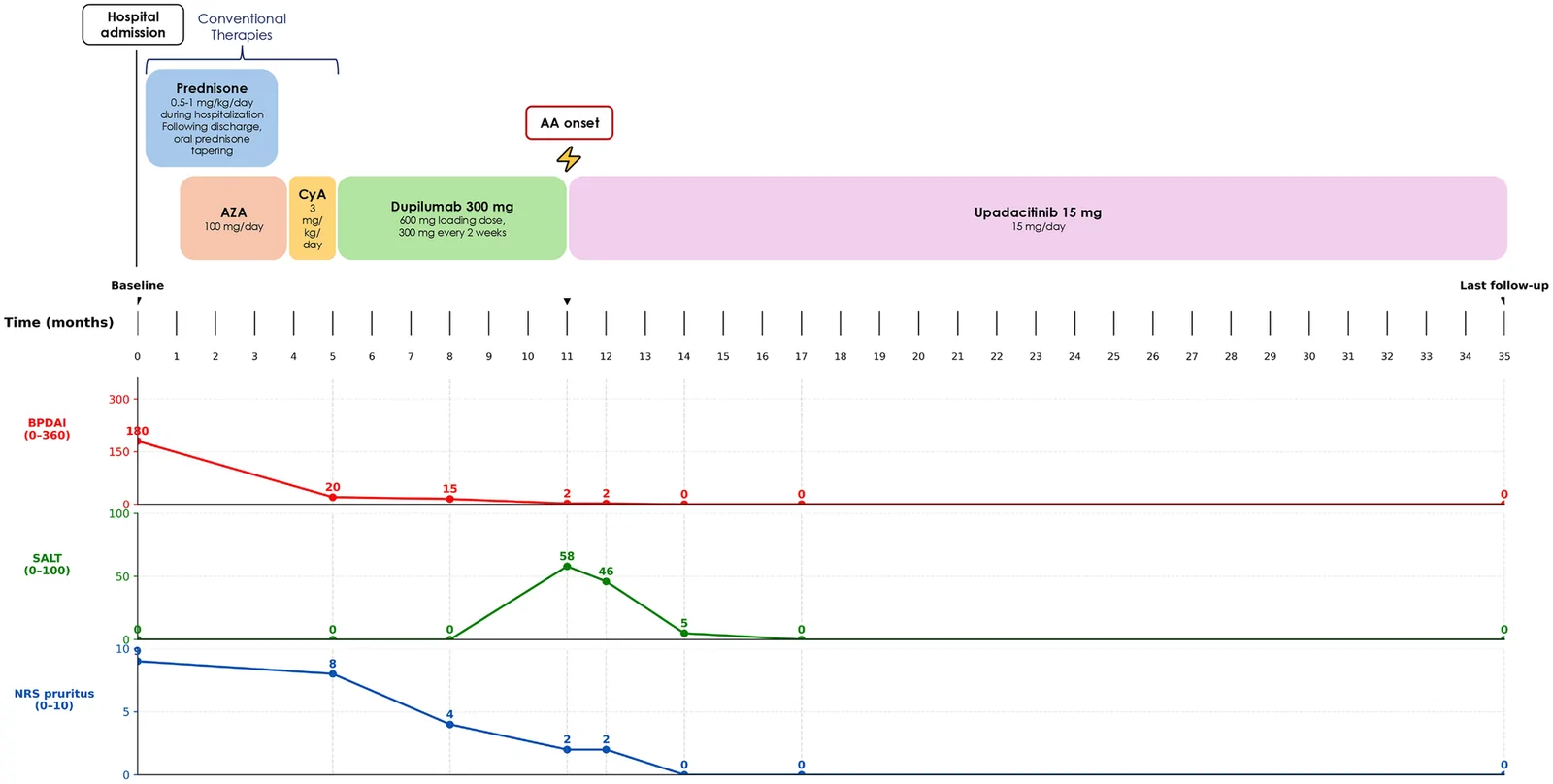
Clinical timeline, treatments, and disease activity over the 35-month follow-up. The upper panel summarizes the patient's therapeutic course, including systemic corticosteroids (prednisone), azathioprine (AZA), cyclosporine (CyA), dupilumab, and upadacitinib. Alopecia areata (AA) developed after 6 months of dupilumab therapy, prompting treatment discontinuation and initiation of upadacitinib. The lower panels illustrate longitudinal changes in Bullous Pemphigoid Disease Area Index (BPDAI) activity score, Severity of Alopecia Tool (SALT) score, and pruritus Numeric Rating Scale (NRS), demonstrating sustained remission during long-term upadacitinib therapy.

**Figure 2:**
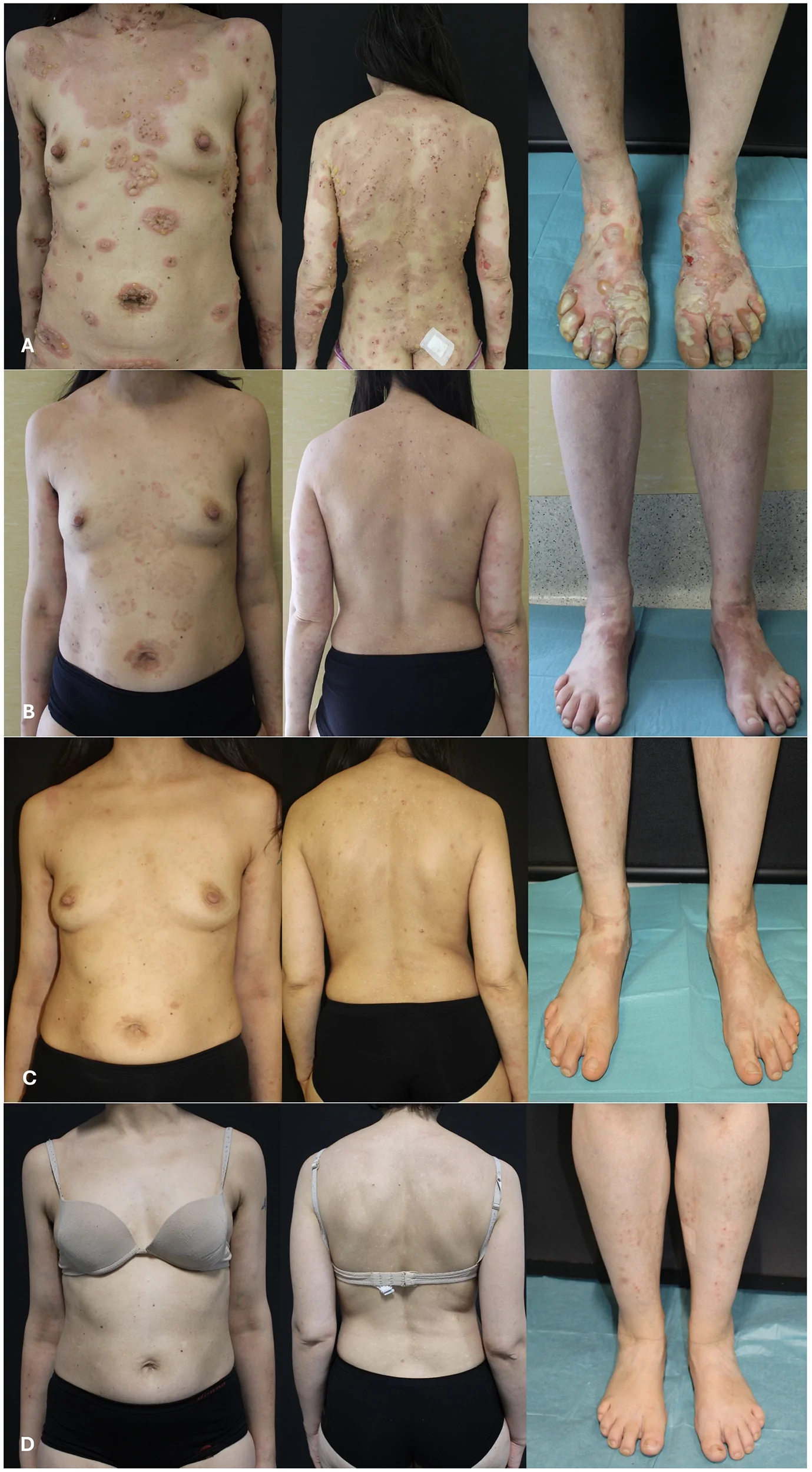
Sequential clinical photographs documenting the evolution of bullous pemphigoid and atopic dermatitis. **(A)** Baseline at bullous pemphigoid diagnosis, before initiation of dupilumab, showing extensive tense bullae and erosions involving the trunk, back, and lower extremities. **(B)** Month 5, immediately before dupilumab initiation (Week 0), demonstrating marked improvement following systemic corticosteroids and conventional immunosuppressive therapy, with residual active disease. **(C)** Month 11, at dupilumab discontinuation (Week 24) and initiation of upadacitinib (Week 0), showing complete remission of bullous pemphigoid and atopic dermatitis. **(D)** Month 35, after 24 months of upadacitinib therapy, demonstrating sustained complete remission without clinical relapse.

**Figure 3:**
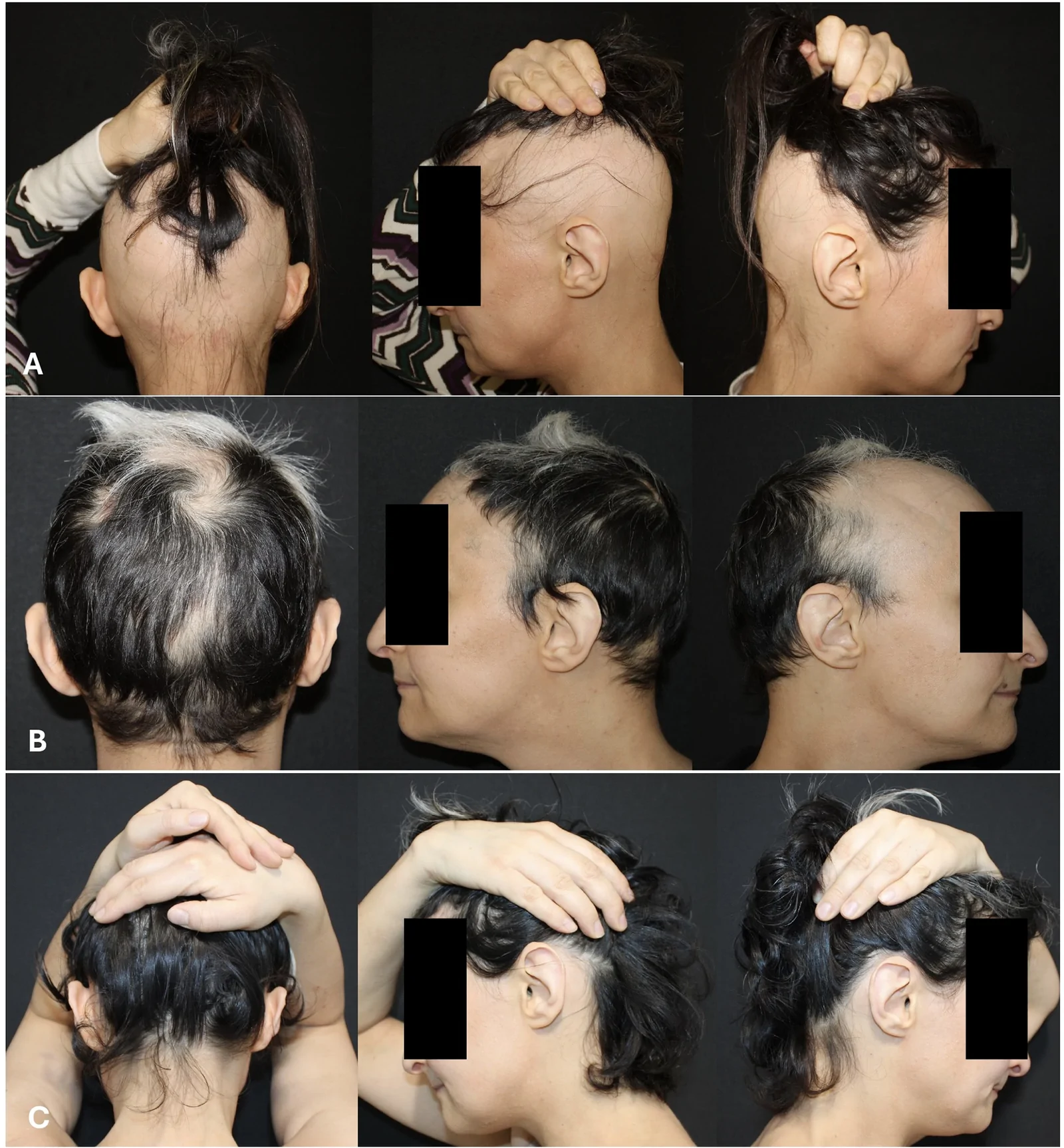
Sequential clinical photographs documenting the evolution of alopecia areata. **(A)** Month 11, at dupilumab discontinuation (Week 24) and initiation of upadacitinib (Week 0), showing new-onset patchy alopecia areata (SALT 58). **(B)** Month 14, after 12 weeks of upadacitinib therapy, demonstrating marked hair regrowth (SALT 5). **(C)** Month 35, after 24 months of upadacitinib therapy, showing complete hair regrowth with sustained remission (SALT 0).

## 3 Discussion

This case highlights the interplay of immune pathways in a patient with three immune-mediated dermatologic diseases: AD, BP, and AA. The coexistence of AD and BP is unusual, while the subsequent onset of AA during dupilumab therapy further underscores the immunologic complexity. The case illustrates how targeted immune modulation may shift the balance between inflammatory pathways, contributing to additional immune-mediated diseases, whereas therapeutic strategies targeting shared intracellular signaling pathways may help re-establish immune equilibrium. AD and BP share a prominent Th2 immune axis (2, 5). Notably, our patient had a longstanding atopic background, including asthma, allergic rhinitis, and elevated IgE levels. Epidemiologic studies have shown that a history of AD confers an increased risk of developing BP compared with non-atopic individuals, even after adjustment for age and comorbidities (5, 12). These findings support the hypothesis that chronic type-2 inflammation and skin barrier dysfunction in severe AD may create an immunologic milieu permissive to the development of BP autoimmunity. In contrast, AA is classically considered a Th1-driven autoimmune disease, in which collapse of the hair follicle immune privilege is thought to be driven by interferon-*γ*–producing CD8⁺ T cells and NKG2D⁺ cytotoxic cells. Nevertheless, AA represents a heterogeneous entity. In the context of AD, AA may be interpreted not only as a distinctive autoimmune disorder, but also as a potential dermatologic comorbidity arising within a broader systemic inflammatory background (13).

Recent analyses of dupilumab in AD patients with coexisting AA support different immune endotypes, including Th1- and Th2-skewed forms (3). Starace et al. observed that worsening of AA on dupilumab occurred mainly in young males with patchy alopecia, suggesting a Th1-driven profile in which Th2 inhibition may shift the balance toward Th1 pathways and enhance the autoimmune response against hair follicles (3). Conversely, patients, often female, with more extensive or longstanding alopecia tended to improve, possibly reflecting a greater Th2 contribution (3). This “dual effect” of dupilumab in AA has been termed a *Janus effect*, reflecting opposite outcomes depending on the immunologic milieu (14). In our case, the development of patchy AA six months after initiation of dupilumab suggests a possible Th1-mediated process following Th2 pathway inhibition. Although a coincidental occurrence cannot be excluded, the temporal association and consistency with previous reports support a potential link, particularly in individuals with a predisposing immune background. This case underscores the need for clinical vigilance regarding unexpected immune-mediated phenomena in complex patients. The complexity of overlapping immune pathways also has therapeutic implications. Targeting the JAK–STAT pathway allows modulation of multiple inflammatory axes, including both Th1- and Th2-driven responses. JAK inhibitors are already approved for AD and AA, whereas their use in BP remains investigational. Nevertheless, emerging clinical reports support the potential efficacy of JAK–STAT inhibition in BP, particularly in refractory or complex cases characterized by overlapping inflammatory diseases (2, 15, 16). Within this framework, switching to upadacitinib resulted in rapid and sustained control of all three conditions. Following achievement of remission, clinical remission of AD, BP, and AA was sustained throughout the twenty-four-month follow-up. Notably, treatment was well tolerated, with no significant safety concerns observed, supporting the favorable safety profile of JAK1 inhibition in this clinical setting.

To better contextualize our findings, we reviewed the available literature on upadacitinib in patients with multiple coexisting immune-mediated dermatologic diseases (Table 1). Although evidence is limited to case reports and small series, upadacitinib has shown clinical benefit across overlapping conditions, including AD, AA, BP, vitiligo, psoriasis, and hidradenitis suppurativa, often after failure or intolerance of conventional systemic therapies and/or biologics. Treatment was generally well tolerated, with acne as the most frequently reported adverse event and no major safety concerns during follow-up. The present case is notable for the sustained remission of three coexisting dermatologic diseases and the relatively long follow-up.

**Table 1:** Clinical characteristics, treatment, and outcomes of published case reports on upadacitinib in patients with multiple immune-mediated dermatologic diseases.

Case	Age/ Gender	Dermatologic IMIDs	Other IMIDs	Prior therapies	Concomitant therapies	Upadacitinib regimen	FU	Outcomes	Adverse effects
Gambardella et al., 2021 (case 1) (17)	30/M	AD, AA	-	CyA, dupilumab	None	30 mg	4 mo	CR	None
Gambardella et al., 2021 (case 2) (17)	42/F	AD, AA	Asthma	nb-UVB, CyA, AZA, dupilumab	None	30 mg	4 mo	CR	None
Pan et al., 2023 (18)	16/M	AD, vitiligo	None	CCT	Crisaborole	15 mg	7 mo	PR	Acne
Novielli et al., 2024 (19)	28/M	AD, AA	CD	CyA, AZA, dupilumab	None	30→15 mg	9 mo	CR AD, PR AA	Acne
Cantelli et al., 2022 (20)	24/M	AD, AA	-	CCT, CCS, CyA, dupilumab	None	30 mg	3 mo	CR	None
Muzy, 2024 (21)	27/M	AD, AA	-	MTX, phototherapy	None	15 mg	8 mo	CR	None
Mu et al., 2024 (22)	9/F	AA, vitiligo	-	CCS, TCI	nb-UVB	15 mg	7 mo	PR	None
Su et al., 2024 (23)	66/M	BP, PsO	-	CCS, Dupilumab	None	15 mg	3 mo	CR	None
Tsunoda et al., 2024 (24)	25/M	AD, PsO	PsA	Dupilumab, CyA, ixekizumab, CCS, baricitinib	None	30 mg	4 mo	CR	None
Hong Yiu et al., 2024 (25)	32/F	AA, PsO	UC, Graves disease, Addison disease	Infliximab, mercaptopurines	None	45→30 mg	4 mo	CR	None
Giavina-Bianchi and Giavina-Bianchi, 2024 (26)	29/M	AD, AA	Asthma	MTX, CyA, CCS	None	30 mg	9 mo	PR	Acne
Levine et al., 2025 (27)	20/M	AA, AD	CD	Adalimumab	Oral minoxidil	45→30 mg	8 mo	CR	None
Okazaki et al., 2025 (28)	69/M	PsO, AD	PsA	Brodalumab, risankizumab, CyA, dupilumab	None	15 mg	24 mo	PR	None
Magnanimi et al., 2025 (29)	25/F	AD, vitiligo	-	CCS, CCT, TCI, CyA	None	15 mg	12 mo	CR AD, PR vitiligo	Acne
Wang et al. 2026 (30)	57/M	AD, vitiligo, AA	-	CCS, CCT, TCI	None	15 mg	20 mo	CR AD, CR AA, PR vitiligo	Hyperpigmentation
Current case	33/F	AD, BP, AA	Celiac disease	CCS, AZA, CyA, dupilumab	None	15 mg	24 mo	CR	None

AA, alopecia areata; AD, atopic dermatitis; AZA, azathioprine; BP, bullous pemphigoid; CCS, systemic corticosteroids; CCT, conventional topical corticosteroids; CD, Crohn's disease; CR, complete remission; CyA, cyclosporine; FU, follow-up; IMIDs, immune-mediated inflammatory diseases; MTX, methotrexate; nb-UVB, narrowband ultraviolet B phototherapy; PR, partial remission; PsA, psoriatic arthritis; PsO, psoriasis; TCI, topical calcineurin inhibitors; UC, ulcerative colitis.

From the patient's perspective, the recurrent blistering and severe pruritus associated with BP had a substantial impact on daily life, while the subsequent development of AA represented an additional concern. Following the switch to upadacitinib, the patient reported progressive improvement in hair regrowth together with sustained control of skin lesions and pruritus. At long-term follow-up, she expressed satisfaction with the simultaneous control of all three conditions with a single, well-tolerated treatment.

Some limitations of this report should be acknowledged. The single-case design precludes generalization and does not allow the establishment of a causal relationship between dupilumab therapy and the onset of AA. In addition, no immunologic investigations were performed to directly characterize immune pathway shifts, and the duration of follow-up remains limited. Despite these limitations, this case adds to the growing body of evidence supporting broader immunomodulatory strategies targeting shared signaling pathways. Further prospective and mechanistic studies are warranted to better define the role of JAK inhibitors in patients with overlapping immune-mediated skin diseases and their long-term efficacy and safety in this challenging clinical setting.

## Data Availability

The raw data supporting the conclusions of this article will be made available by the authors, without undue reservation.
